# Development of a Molecular Imprinting-Based Surface Plasmon Resonance Biosensor for Rapid and Sensitive Detection of *Staphylococcus aureus* Alpha Hemolysin From Human Serum

**DOI:** 10.3389/fcimb.2020.571578

**Published:** 2020-11-20

**Authors:** Tilde Andersson, Anna Bläckberg, Rolf Lood, Gizem Ertürk Bergdahl

**Affiliations:** ^1^Division of Infection Medicine, Department of Clinical Sciences Lund, Lund University, Lund, Sweden; ^2^Department of Infection Medicine, Skåne University Hospital, Lund, Sweden

**Keywords:** sepsis, *Staphylococcus aureus*, alpha hemolysin, molecular imprinting, SPR

## Abstract

*Stapylococcus aureus* is a common infectious agent in *e.g.* sepsis, associated with both high mortality rates and severe long-term effects. The cytolytic protein α-hemolysin has repeatedly been shown to enhance the virulence of *S. aureus*. Combined with an unhindered spread of multi drug-resistant strains, this has triggered research into novel anti virulence (*i.e.* anti α-hemolysin) drugs. Their functionality will depend on our ability to identify infections that might be alleviated by such. We therefore saw a need for detection methods that could identify individuals suffering from *S. aureus* infections where α-hemolysin was a major determinant. Molecular imprinted polymers were subsequently prepared on gold coated sensor chips. Used in combination with a surface plasmon resonance biosensor, α-hemolysin could therethrough be quantified from septic blood samples (n = 9), without pre-culturing of the infectious agent. The biosensor recognized α-hemolysin with high affinity (K_D_ = 2.75 x 10^-7^ M) and demonstrated a statistically significant difference (*p* < 0.0001) between the α-hemolysin response and potential sample contaminants. The detection scheme proved equally good, or better, when compared to antibody-based detection methods. This novel detection scheme constitutes a more rapid, economical, and user-friendly alternative to many methods currently in use. Heightening both reproducibility and sensitivity, molecular imprinting in combination with surface plasmon resonance (SPR)-technology could be a versatile new tool in clinical- and research-settings alike.

## Introduction

Although commonly isolated as an asymptomatic colonizer, *Staphylococcus aureus* is also the leading cause of blood- (Pfaller et al., 1999), skin- and soft tissue-infections (Fridkin et al., 2005) worldwide. The rapid spread of multidrug resistant strains, now endemic in many parts of the world (Diekema et al., 2001), is making treatment increasingly challenging and prompting research into novel anti-virulence drugs (Kane et al., 2018). Numerous virulence factors, with functions ranging from modification of neutrophil responses (Thammavongsa et al., 2013) and complement activation (Rooijakkers et al., 2005) to host cell lysis (Powers et al., 2012), are secreted during *S. aureus* infections. The latter, a ß-barrel-forming cytotoxin termed α-hemolysin (α-toxin, Hla), is heavily involved in the pathogenesis of sepsis due to *e.g.* its negative effect on endothelial integrity (Powers et al., 2012). Produced to some degree by approximately 80–99% of clinical isolates (Tabor et al., 2016), α-hemolysin expression has furthermore been shown to correlate with virulence (Jenkins et al., 2015). The performance of future anti-virulence drugs depends on our ability to identify infections that would be alleviated by such. We consequently saw need for a novel detection method that could rapidly, with high sensitivity and selectivity, recognize patients suffering from an *S. aureus* infection where α-hemolysin is a major determinant.

Current guidelines state that for any suspected *S. aureus* infection, almost irrespective of the infection site/syndrome, samples should be taken for culturing (David and Daum, 2017). The sensitivity of culture-based systems is generally low (Vincent et al., 2006), meaning that for septic patients, where delayed or sub-optimal treatment is associated with increased mortality (Josefson et al., 2011), culture-based systems struggle to provide accurate results within a clinically relevant time-frame (Gaibani et al., 2009). Comparatively, nucleic acid based tests are generally quicker and could provide not only a precise pathogen identification, but also antimicrobial susceptibility profiles (Lehmann et al., 2008). Other alternatives include Western blots, ELISA systems, and/or MALDI-TOF-MS (Song et al., 2017). Although arguably more sophisticated, these methods raise different concerns in the form of repeatability, throughput, total cost, and the risk of detecting overall DNAemia rather than solely the pathogenic agent of interest. We therefore suggest a novel surface plasmon resonance (SPR)-based detection scheme in which *S. aureus* α-hemolysin can be quantified directly from patient samples, within a matter of minutes, eliminating the need for intermediate processing.

The popularity of biosensors, in particular those based on SPR-technology, has drastically increased since they were first introduced in the 1980’s (Liedberg et al., 1983). They couple the use of biological components, such as proteins or nucleic acids, with an optical signal transducer to relay information about affinity, concentrations and binding kinetics (Situ et al., 2010). Biosensor applications range from clinical (Gupta et al., 2012) and pharmaceutical (Cooper, 2002) to biological warfare detection (Gupta et al., 2010). While most studies make use of the inherent binding between an antibody and its antigen, we elected to combine SPR technology with imprinting techniques. A molecular imprinted polymer (MIP) consists of a cross-linked polymeric matrix formed around a template molecule of interest (here α-hemolysin). A three-dimensional cavity, complementary to the template in shape, size and placement of functional groups, is created in the polymer (Shea and Sasaki, 1989). These artificially created recognition sites can subsequently re-bind free template molecules with high affinity during, for instance, protein extraction or biomarker detection/quantification (Boysen, 2019). It excludes not only the need for molecular labelling, but also that of antibody manufacturing and hence the use of research animals therein.

In this study, we have established a novel MIP- and SPR-based α-hemolysin detection scheme. Our results suggest that MIP technology could be useful in detection of this diagnostic, prognostic and potentially predictive (*i.e.* response to anti-virulence drugs) biomarker of sepsis.

## Methods

### Ethics

The study was approved by the regional committee in Lund, Sweden (ref. no. 2018/830). Informed written, and oral, consent was obtained from all patients included before enrolment.

### Preparation of α-Hemolysin Imprinted (MIP) and Non-Imprinted (NIP) Chips

A detailed description of MIP chip preparation procedures can be found elsewhere (Ertürk Bergdahl et al., 2019). Briefly, α-hemolysin (H9395, Sigma Aldrich) was dissolved in phosphate buffer (10 mM, pH 7.4) to a concentration of 0.1 mg/ml and immobilized onto a pre-treated glass surface, forming a protein (α-hemolysin) mold. A 1.3 µl aliquot of monomer solution [(N-2-Hydroxyethyl methacrylate, 10 %, v/v, Sigma Aldrich, 245801), (Polyethylene glycol dimethacrylate, 50 %, v/v, Sigma Aldrich, 409510), MQ water (40 %, v/v) and (10 mM 1,1’-Azobis cyclohexanecarbonitrile, Sigma Aldrich, 380210)] was placed on the gold surface of a pre-treated Biacore sensor chip (SIA kit Au, Cytiva). Subsequently placed upon the protein mold, all components received 10 min of UV irradiation (Dymax, 400 W, 365 nm). A non-imprinted (NIP) chip was prepared using the same method but excluding the template protein.

### Preparation of Anti-α-Hemolysin Immobilized Chips

The Amine Coupling Kit (BR100050, Cytiva) containing ethanolamine, *N-*hydroxysuccinimide (NHS) and *N*-ethyl-*N*’-dimethylaminopropylcarbodiimide (EDC), was used throughout the process. The procedure for amine coupling, available on the Biacore x100 instrument (Cytiva), was followed. Anti-α-hemolysin (S7531, Sigma Aldrich) diluted in acetate buffer (10 mM) pH 5.0 was immobilized to a level of 1200 RU.

### Sensitivity Analysis

Prepared chips were calibrated with HBS-EP+ buffer (BR100669, Cytiva), using the Biacore x100 (Cytiva), until a stabile baseline was reached. An α-hemolysin (H9395, Sigma Aldrich) dilution series (0.012-0.76 µM) was prepared in the same buffer and subsequently injected into the system (imprinted and immobilized) in technological triplicates. The NIP chip was used as a negative control for MIP measurements.

In order to analyze the samples with Biacore X100, sample solution is injected over the sensor surface using an autosampler. All steps including surface preparation, binding and regeneration are monitored in a sensorgram which is a plot of response against time, showing the progress of the interaction. Binding is displayed directly on the computer screen during the course of the interaction which makes the whole analysis real-time. The response is measured in resonance units (RU) which is directly proportional to the concentration of biomolecules accumulated on the surface.

### Selectivity Analysis

Solutions containing putative cross-reactants (0.1 mg/ml), including cholera toxin (C8052), IgG (I4506), albumin (H0900000), fibrinogen (F3879), collagen (CC050), and keratin (K0253) (all purchased from Sigma Aldrich), were prepared in HBS-EP+ buffer (BR100669, Cytiva). The solutions were injected into each system (MIP, NIP and the antibody-immobilized) separately, using the Biacore x100 (Cytiva). Response units (RU) were recorded, and the NIP chip was used as a negative control for MIP measurements.

### Sample Collection

Blood samples were collected at Lund University hospital, clinic of infectious diseases, between 2018 and 2019, from patients with confirmed blood infections (*n* = 14). Whole blood was collected into serum tubes before the serum fraction could be manually isolated following centrifugation (1500 g, 10 min). Patient samples that were culture negative for *S. aureus*, but positive for other infectious agents (*n* = 5), were used as negative controls during establishment of calibration curves (**Figure S3**).

### Quantification of α-Hemolysin From Patient Samples

Serum samples collected from the patients with confirmed *S. aureus* infection (n = 9) and control serum samples were initially centrifuged by using 100 K spin columns (88503, Thermo Fischer) to remove the most abundant proteins with MW ≥100 kDa and subsequently diluted 1:10 in HBS-EP+ buffer (BR100669, Cytiva) before being analyzed using the MIP chip, the antibody immobilized chip, and ELISA. Patient samples that were culture negative for *S. aureus*, but positive for other infectious agents were used as negative controls and spiked with alpha hemolysin (0.047–3.03 µM) to generate calibration curves (**Figure S3**) which were then used to calculate serum alpha hemolysin concentrations in patient samples.

### Sandwich ELISA Analysis

A 96-well plate (Nunc, MaxiSorp) was coated O/N, at 4 °C, with 100 µl anti-α-hemolysin (S7531, Sigma Aldrich) diluted 1:12 500 (V/V) in sodium carbonate buffer (0.05 M, pH: 9.5). Wells were then blocked for 2 h, RT, using 250 µl BSA (1 %, W/V) in wash buffer. Patient samples and alpha-hemolysin spiked serum samples, diluted 1:10 (V/V) in PBS, were subsequently added to the wells in duplicates (100 µl/well) and incubated for 2 h. Anti-α-hemolysin (S7531, Sigma Aldrich) diluted 1:12 500 (V/V) were added (100 µl/well) for an additional 2 h before secondary HRP-conjugated goat anti-rabbit-antibodies (Bio-Rad, 1721019), diluted 1:4000 (V/V) in wash buffer, was finally added (1 h, RT). Wells were incubated for 10–15 min with substrate 3,3′,5,5′-Tetramethylbenzidine (TMB, Sigma Aldrich, T4444) (100 µl/well) before stop solution in the form of 100 µl H_2_SO_4_ (1 M) was added and the absorbance was read at 450 nm. Between each steps, wells were washed 3 times using 0.05 % Tween 20 in PBS (V/V). The absorbance values recorded from the alpha-hemolysin spiked serum samples were used to draw a calibration curve which was then used for the quantification of alpha hemolysin in patient samples.

## Results

### Sensitivity and Selectivity of the α-Hemolysin Imprinted (MIP) Chip

**Figure 1:**
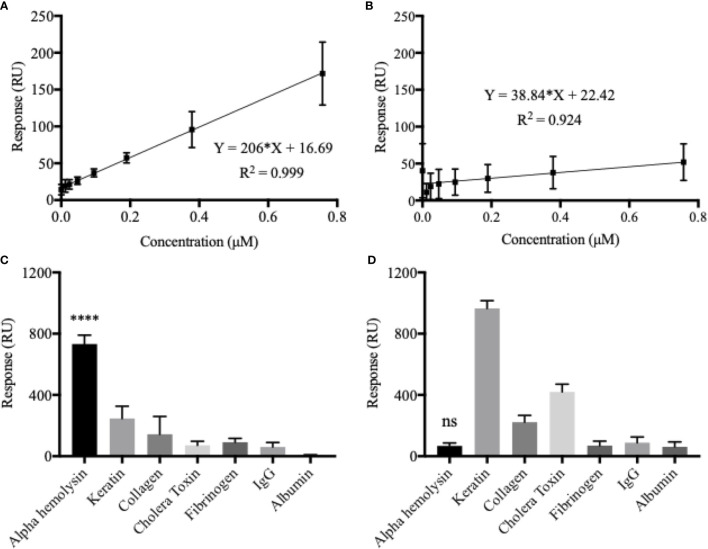
Chip sensitivity and selectivity is heavily dependent on the formation of template-molecular imprints. Calibration curves were established using an α-hemolysin dilution series of 0.012–0.76 µM. The molecular imprinted polymer (MIP) **(A)** yielded a considerably higher response and showed a much broader dynamic range as compared to the non-imprinted polymer (NIP) **(B)**. The MIP essentially demonstrated a double binding response (RU) for every doubling in α-hemolysin concentrations. Although some unspecific binding, low for potential blood-sample contaminants but higher for potential skin-swab contaminants, could be detected, the MIP proved to be highly selective for α-hemolysin **(C)**. The NIP showed no α-hemolysin selectivity **(D)**. All presented values represent the mean of triplicates with corresponding SD values. One-way ANOVA analysis was used to evaluate selectivity results **(C**, **D)**. ****P < 0.0001; ns, non-significant.

An α-hemolysin dilution series, with concentrations ranging between 0.012–0.76 µM, were injected into the MIP system using Biacore x100. A calibration curve, with a high degree of fit (R^2^ = 0.999), could subsequently be created (**Figure 1A**). Data from this experiment confirmed that the biosensor was able to recognize the template molecule with high affinity (K_D_ = 2.75 x 10^-7^ M), and when a saturation curve was established, the maximum response was strong, around 600 RUs (**Figure S1**). Analysing the formerly described dilution series with the non-imprinted (NIP) chip, left the intensity of the response markedly lowered (**Figure 1B**). Limit of detection (LOD) values were calculated from the respective formulas (3xSD from blank measurements) and found to be 0.022 and 2.256 µM for the MIP- and NIP-chip respectively. These results illustrate how, although some unspecific binding to the polymer surface can be detected, template imprinting is crucial for accurate and uniquely determined measurements to be made.

Selectivity of the system was analyzed in terms of the MIP chip’s interaction with potential cross-reactants, *i.e.* other proteins that might be found in blood samples such as IgG, fibrinogen and albumin. Since the system could potentially also be used for other types of *S. aureus* infections, such as skin, additional possible contaminants, such as keratin and collagen were also tested for selectivity (**Figure 1C**). Cholera toxin was included to ensure that structural similarity between analyte and template protein was not enough to cause noteworthy interaction with the MIP. When all analytes were added at a concentration of 0.1 mg/ml, α-hemolysin generated the greatest response by far (732.98 RU), followed by keratin (mean difference 486.4 RU or 66.4%) and collagen (mean difference 589.5 RU or 80.4%). A one-way ANOVA analysis confirmed statistically significant differences between the MIP’s response to α-hemolysin, compared to all potential cross reactants (*p* < 0.0001). It is clear from the selectivity profile of the NIP chip (**Figure 1D**), that a lack of artificially created recognition sites left the system without any selectivity for α-hemolysin (*p* > 0.05 or higher RU for cross-reactants).

### Sensitivity and Selectivity of the Anti-α-Hemolysin Immobilized Chip

**Figure 2:**
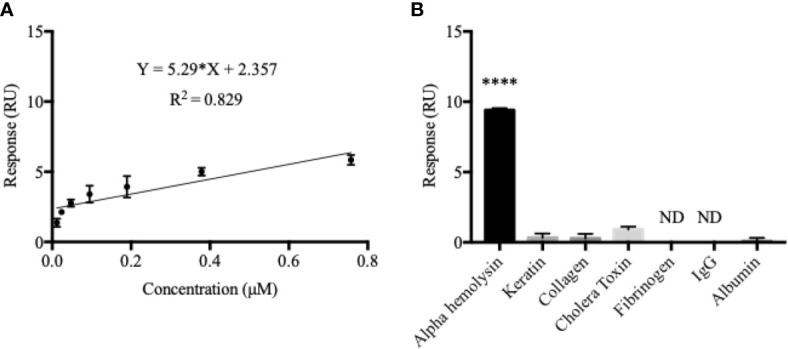
The antibody-immobilized chip generated a sub-optimal calibration curve but displayed a better α-hemolysin selectivity. The calibration curve for the antibody-immobilized chip was established in the same way as for the MIP **(A)**. Here, a double response (RU) was not seen as a result of doubling in α-hemolysin concentrations which effectively decreased both the dynamic range and the degree of fit (R^2^ = 0.829). Contrarily, the antibody-immobilized chip proved to be highly selective for α-hemolysin, with little or no response recorded for all potential blood-sample contaminants **(B)**. Values are presented as the mean of triplicates with standard deviations. A one-way ANOVA analysis was used to evaluate selectivity results. ****P < 0.0001; ND, Non-Detectable.

In order to compare the MIP-based detection system to one which is more commonly used in combination with SPR-technology today, an anti-α-hemolysin antibody was immobilized onto a commercially available Biacore sensor chip. A calibration curve was established by analyzing the same α-hemolysin dilution series as before (0.012–0.76 µM) using the new chip (**Figure 2A**). Compared to that of the imprinted chip, degree of fit was less satisfying (R^2^ = 0.829), affinity was similar (K_D_ = 3.21 x 10^-7^ M), and LOD was 10 times better (0.002 µM) for the anti-α-hemolysin immobilized chip. The saturation curve generated for the immobilized system showed a slightly higher R^2^ value but saturation with a much lower maximum response (**Figure S2**). Even though it displayed better sensitivity, as indicated by the low LOD value, the antibody immobilized system reached saturation at lower concentrations, suggesting that the MIP chip might perform better in clinical settings where a wider dynamic range is preferable.

The antibody immobilized system proved furthermore to be highly selective for α-hemolysin (**Figure 2B**). The response triggered by α-hemolysin (9.40 RU) was much greater than, yet followed by, cholera toxin (mean difference 8.5 RU or 90.4%) and keratin (mean difference 9.07 RU or 96.5%). Responses for fibrinogen and IgG were non-detectable (ND). Like in the MIP chip measurements, a one-way ANOVA analysis confirmed statistically significant differences between the immobilized chip’s response to α-hemolysin compared to all potential cross reactants (*p* < 0.0001). For easy comparison, selectivity coefficients (response α-hemolysin/response cross-reactant) were established for all chips (MIP, NIP and the antibody-immobilized chip) (**Table 1**).

**Table 1:** With the exception of albumin, the antibody-immobilized chip showed greater selectivity coefficients for all potential cross-reactants.

Analyte	Response (RU) _MIP chip_	*K*_(MIP chip)_	Response (RU) _NIP chip_	*K*_(NIP chip)_	Response (RU) _Immobilized chip_	*K*_(Immobilized chip)_
**Alpha hemolysin**	733.0	–	67.82	–	9.4	–
**Keratin**	246.6	2.97	965.4	0.07	0.3267	28.77
**Collagen**	143.5	5.10	222.7	0.30	0.2833	33.18
**Cholera Toxin**	71.44	10.26	420.2	0.16	0.9	10.44
**Fibrinogen**	91.21	8.04	69.1	0.98	ND	ND
**IgG**	59.49	12.32	88.44	0.77	ND	ND
**Albumin**	4.723	155.20	61.29	1.10	0.08	117.50

No interaction could be detected between the immobilized chip and fibrinogen or IgG. Interactions with potential skin-swab contaminants were also lower (generating a higher selectivity coefficient). Only albumin had a percentually lower binding to the MIP. Selectivity coefficients (K) were calculated using: Response _(α-hemolysin)_/Response _(cross-reactant)_.

### Quantification of α-Hemolysin Directly From Patient Samples

**Figure 3:**
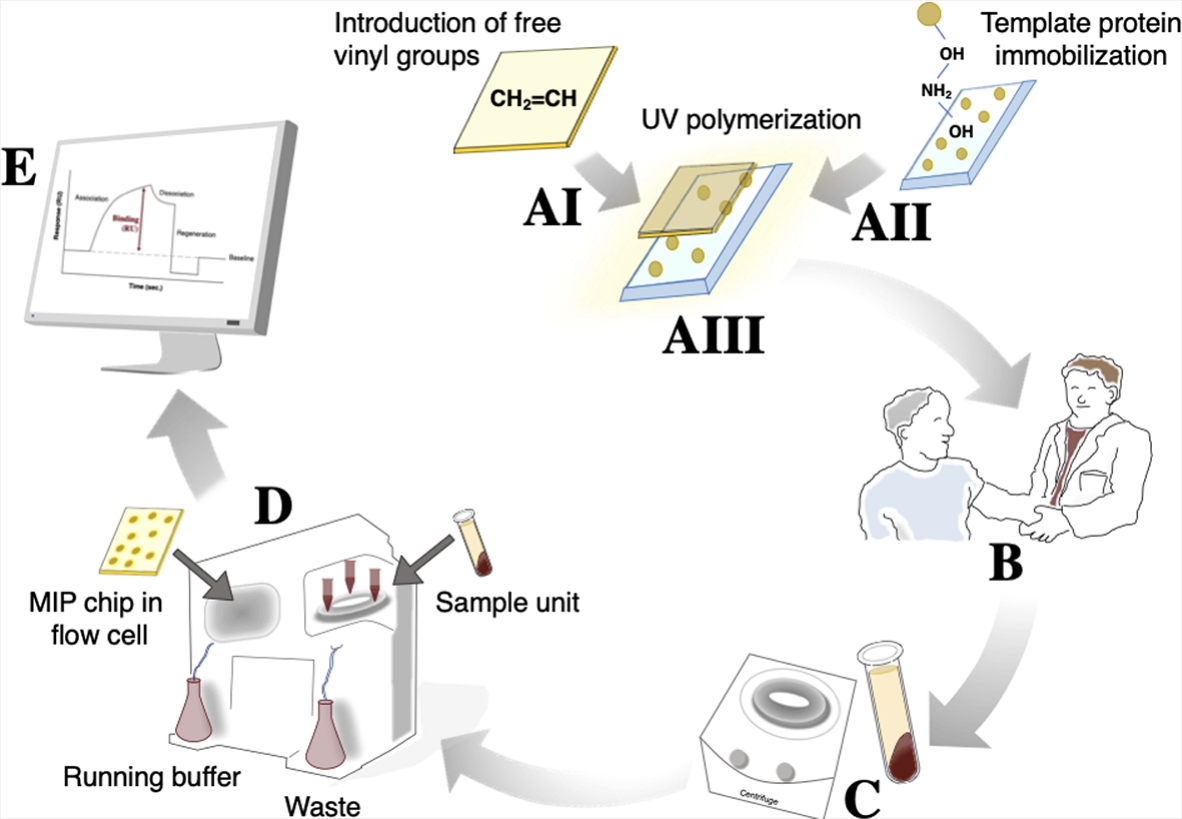
The procedure used for α-hemolysin analysis from patient samples includes 5 main steps. The initial MIP chip construction **(A)** can be divided further into gold chip surface preparation for introduction of free vinyl groups (AI), glass surface treatment for subsequent template protein immobilization (AII), and UV polymerization around the immobilized template protein (AIII). Because the MIP is stabile for month/years if stored under the right conditions, (AIII) can take place long before actual use. Once a blood sample is drawn **(B)**, coagulation and a 10 min centrifugation **(C)** will enable separation of serum. Diluted serum can subsequently be injected into the MIP/SPR-system **(D)** from which results can be read directly, and potentially sent to the treating physician, within 30 min of blood being drawn **(E)**.

**Figure 4:**
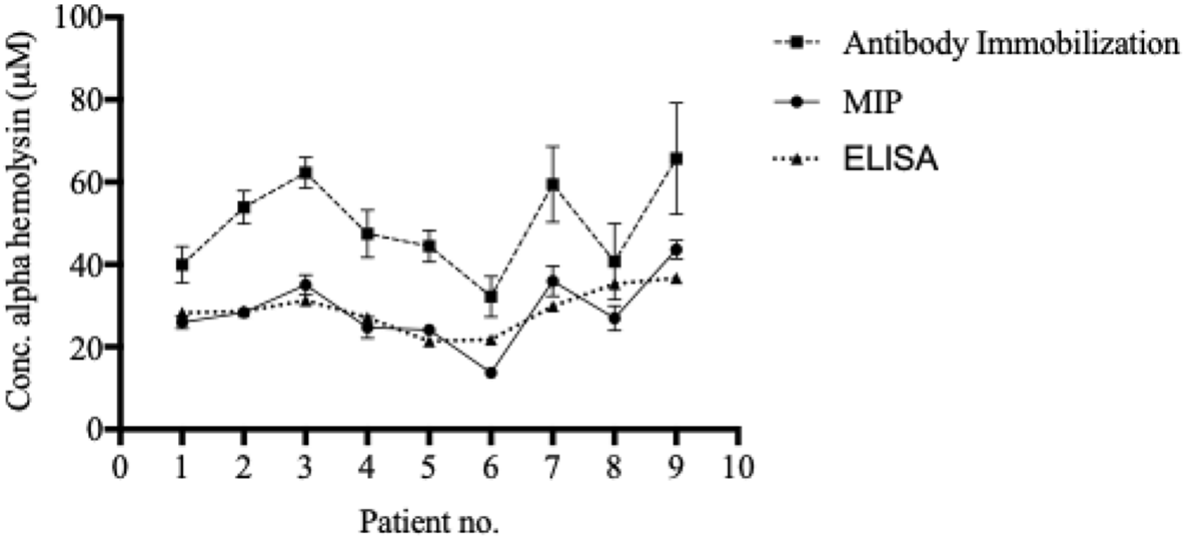
Blood α-hemolysin levels can be determined using MIP- and SPR-technology. Serum was separated from whole blood and diluted 1:10 in HBS-EP+ buffer before injection into Biacore x100. Both the MIP, the immobilized system, and the ELISA generated comparable, and similarly ranking, values. Values in are presented as the mean of triplicates with standard deviations.

The MIP chip, demonstrating good sensitivity (especially at high α-hemolysin concentrations) and selectivity (especially for albumin, the most abundant protein in blood), was subsequently used for direct quantification of α-hemolysin in patient samples (**Figure 4**). A schematic representation of the procedure used is shown in **Figure 3**. Serum samples were collected from individuals with systemic bacterial infections (*n* = 14) and diluted 1:10 in HBS-EP+ buffer before analysis. Those that were culture positive for *S. aureus* (*n* = 9) were termed 1–9, while non- *S. aureus* positive infections (*n* = 5) were used as negative controls in the making of calibration curves. Serum α-hemolysin concentrations could be directly extracted from patient data by using the equation presented in the calibration curves in **Figure S3**. As the procedure was not culture based, these quantifications were related to the level of toxin in patient blood, rather than the bacterial strain’s expression of toxin *in vitro*. High serum alpha hemolysin concentrations seemed furthermore to be correlative with increased mortality rates. Within the high scoring half, and the low scoring half, of the samples tested, mortality rates were 40 and 0% respectively.

To verify the accuracy of the patient sample alpha hemolysin concentrations calculated by using the MIP chip, both the anti-alpha hemolysin immobilized chip and the ELISA system were used to quantify serum alpha hemolysin concentrations in the same samples. While the biosensor recorded slightly higher values for each sample when the antibody immobilized chip was used, the trend (the individual ranking of patients) was similar to the MIP chip (**Figure 4**). The ELISA analysis gave values highly similar to those recorded using the MIP chip (**Figure 4**).

## Discussion

Here, we designed a novel α-hemolysin detection scheme and evaluated its potential usability as a tool for (primarily) clinical testing. Enabling rapid diagnosis of alpha hemolysin expressing *S. aureus* strains in critically ill patients, results were generated in only minutes (< 0.5 h) after sampling. We purpose that this technology, used in combination with *e.g.* anti-virulence drugs, could constitute an important new tool in both clinical and research settings.

Today, antibodies are widely used in both basic and applied medical sciences, notably also in combination with SPR-based systems. Although many display K_D_-values between 10^-6^–10^-9^ M (Liu et al., 2015), production of high affinity antibodies against poorly immunogenic antigens remain a big challenge, and batch-to-batch variations are frustratingly common (Couchman, 2009). The MIP chip used in the course of this study had a K_D_ value of 2.75x10^-7^ M and a LOD value of 0.022 µM. As such, the α-hemolysin affinity was well within the range of normal antibody-antigen interaction, and the detection limit was 1000 times lower than the recorded bottom patient value, which confirms its usability in clinical settings. Previous research has shown that MIP techniques can instead be combined with capacitive biosensors to reach a detection limit of 2.5x10^-19^ M (Ertürk et al., 2018). However, as α-hemolysin concentrations that low are unlikely to be of any clinical significance, the increased robustness of the user-friendly SPR biosensor is here preferable.

In a previous study, comparing the reproducibility of an antibody-immobilized system to that of an imprinted one, it was found that the former began to lose its detection ability after 25 sample injections while the latter lasted twice that amount (Erturk et al., 2015). Additionally, using a MIP surface eliminates the risk of the chip becoming a target for secreted bacterial enzymes, such as hydrolases, present in the media/sample.

The polymer composition used for MIP chip preparation was chosen based on detailed former characterization and optimization studies, specifically in terms of functionality (sensitivity and selectivity) and on nanosized topographical differences (then assessed using cyclic voltammetry, atomic force microscopy, and scanning electron microscopy) (Ertürk Bergdahl et al., 2019). However, unspecific binding to the polymer surface could still be detected. Both the potential blood- and skin-sample contaminants showed a stronger percentual binding to the MIP chip as compared to the antibody-immobilized chip. Albumin, being the most abundant protein in blood plasma by far (Moman and Varacallo, 2020), was the only exception. Though, when all disadvantages of antibodies, such as short half-life, high cost, use of animals in production and subsequent batch-to-batch variations, are taken into consideration, the MIP system again appear preferable.

More rigorous evaluation, including more patient samples, is required for actual implementation of the scheme into clinical settings. However, this study demonstrates how the use of MIP and SPR based technology could allow physicians to make informed decisions regarding treatment much earlier, as they would not have to wait for the result of bacterial culturing. This, in turn, could help reduce the use of broad-spectrum antibiotics (frequently administered early on) and consequently the spread of antimicrobial resistance (Rhee et al., 2020).

Previously linked to increased virulence (Jenkins et al., 2015), and here linked to increased mortality rates, blood α-hemolysin concentrations also appeared to serve as a prognostic biomarker of sepsis. Despite not including anti-virulence drugs in this study, we speculate that α-hemolysin could also serve as a predictive biomarker (*i.e.* response to anti-α-hemolysin drugs) and hope to test this hypothesis at a later stage. Should adaptation to larger-scale analysis be made, in which more controls and anti-virulence drugs were included, it is likely that the performance of the scheme should improve further.

Our results suggest that MIP technology could be useful in detection of this diagnostic, prognostic and potentially predictive biomarker of sepsis. The detection scheme is inexpensive, robust, easily carried out, and easily manipulated.

## Data Availability Statement

The original contributions presented in the study are included in the article/**Supplementary Material**. Further inquiries can be directed to the corresponding author.

## Ethics Statement

The study was approved by the regional committee in Lund, Sweden (ref. no. 2018/830). Informed written, and oral, consent was obtained from all patients included before enrolment. The patients/participants provided their written informed consent to participate in this study.

## Author contributions

TA performed experiments, analyzed data, and drafted the manuscript. AB assisted with sample collection and processing. RL assisted in study design, manuscript formatting, and offered continuous advice throughout. GEB assisted in study design, manuscript formatting and performed experiments. All authors contributed to the article and approved the submitted version.

## Conflict of Interest

The authors declare that the research was conducted in the absence of any commercial or financial relationships that could be construed as a potential conflict of interest.
