# Supplementary material for: Development of a Molecular Imprinting-Based Surface Plasmon Resonance Biosensor for Rapid and Sensitive Detection of Staphylococcus aureus Alpha Hemolysin From Human Serum
Source: Front Cell Infect Microbiol. 2020 Nov 20;10:571578. doi: 10.3389/fcimb.2020.571578 (PMC7715021; doi:10.3389/fcimb.2020.571578)
Supplement: Supplementary file 1 [file DataSheet_1.docx]

# Development of a molecular imprinting-based surface plasmon resonance biosensor for rapid and sensitive detection of *Staphylococcus aureus* alpha hemolysin from human serum

Tilde Andersson^1^, Anna Bläckberg^1,2^, Rolf Lood^1^, Gizem Ertürk Bergdahl^1#^

^1^Division of Infection Medicine, Department of Clinical Sciences Lund, Lund University, Lund, Sweden

^2^Skåne University Hospital, Department of Infection Medicine, Lund Sweden,

^#^To whom correspondence should be addressed:

Department of Clinical Sciences Lund

Division of Infection Medicine

Biomedical Center B14

Lund University

SE-221 84 Lund
Sweden

gizem.erturk_bergdahl@med.lu.se

Key words: Sepsis, *Staphylococcus aureus*, alpha hemolysin, imprinting (MIP), biosensor

**Figure S1.** **The MIP chip’s saturation curve is indicative of a broad dynamic range.** The curve, generated using an α-hemolysin dilution series of 0.012–12.12 µM, shows a high degree of fit and a broad dynamic range (A). Here, all presented values represent the mean of triplicates with corresponding SD values. An example of an observed sensorgram, for one of these triplicate measurements, is shown in (B). Here, the observed baseline drift occurs between cycles and is consequently corrected for through calculation of binding-responses (binding RU – baseline RU).

**Figure S2. The antibody-immobilized chip’s saturation curve has a high degree of fit, but a narrow dynamic range.** The curve, generated using an α-hemolysin dilution series of 0.012–3.030 µM, had an apparent saturation at lower α-hemolysin concentrations than the MIP chip. Additionally, a narrower dynamic range can here be observed. All presented values represent the mean of triplicates with corresponding SD values.

**Table S1.** **Quantification of α-hemolysin from patient serum yielded values between 13.69 and 43.67 µM.** All samples were run in triplicates before a mean value was extrapolated and converted to concentration units using the equation presented in figure S3. Unspecific binding was accounted for in the calibration curve made in diluted patient serum (figure S3).

| **Patient no.** | **α-hemolysin (1^st^)**  **(𝞵M)** | **α-hemolysin (2^nd^)**  **(𝞵M)** | **α-hemolysin (3^rd^)**  **(𝞵M)** |
| --- | --- | --- | --- |
| 1 | 25,27866 | 24,82843 | 27,66367 |
| 2 | 27,38623 | 28,55926 | 29,19688 |
| 3 | 32,29983 | 36,26917 | 36,61962 |
| 4 | 24,13483 | 22,48479 | 27,59065 |
| 5 | 22,49939 | 24,76515 | 25,22998 |
| 6 | 13,44366 | 13,85495 | 13,76734 |
| 7 | 34,09589 | 33,52641 | 40,19713 |
| 8 | 26,33731 | 24,17133 | 30,24337 |
| 9 | 44,77245 | 40,99051 | 45,23485 |

**A**

**B**

**C**

**Figure S3.** **Calibration curves were established for (A) the MIP chip, (B) the immobilized chip, and (C) the ELISA, using patient control serum diluted 1:10.** All samples were run in triplicates before mean values and standard deviations were plotted. R^2^ values are shown underneath the equations.
